# Effect of pregnancy on scleroderma progression

**DOI:** 10.1177/23971983221101311

**Published:** 2022-06-19

**Authors:** Siobhan Deshauer, Mats Junek, Murray Baron, Karen A Beattie, Margaret J Larché

**Affiliations:** 1 Division of Rheumatology, Department of Medicine, McMaster University, Hamilton, ON, Canada; 2 Division of Rheumatology, Jewish General Hospital, Department of Medicine, McGill University, Québec, QC, Canada

**Keywords:** Pregnancy, systemic sclerosis, disease progression

## Abstract

**Objective:**

To explore the trajectory of scleroderma disease activity in women who experienced a pregnancy after systemic sclerosis diagnosis compared to nulliparous women.

**Methods:**

We analyzed data from the Canadian Scleroderma Research Group registry by identifying nulliparous women and women with ⩾1 pregnancy after systemic sclerosis diagnosis. Patient characteristics were compared between groups at registry entry. Controlling for age, smoking, and time since systemic sclerosis diagnosis, generalized estimating equations tested the effect of pregnancy on force vital capacity, diffusing capacity of the lungs for carbon monoxide, right ventricular systolic pressure, glomerular filtration rate, antibody status, active digital ulcers, physician global assessment of activity, and severity over 9 years.

**Results:**

At registry entry, numbers of women in the nulliparous and pregnancy after systemic sclerosis diagnosis groups were 153 and 45, respectively. Corresponding numbers at 6 and 9 years were 48 and 21, and 18 and 9, respectively. The prevalence of anti-topoisomerase positivity was 18.3% in nulliparous and 12.5% in pregnancy after systemic sclerosis diagnosis. Baseline differences included mean (Standard deviation) age of diagnosis (nulliparous: 38.8 (14.0), pregnancy after systemic sclerosis diagnosis: 22.6 (6.8) years, *p* < 0.001), disease duration (nulliparous: 9.6 (8.9), pregnancy after systemic sclerosis diagnosis: 21.9 (9.6) years; *p* < 0.001), and inflammatory arthritis (nulliparous: 41 (28%), pregnancy after systemic sclerosis diagnosis: 22 (49%), *p* = 0.009). There were no significant differences between groups in the change of any outcomes over time.

**Conclusion:**

Results demonstrated that having ⩾1 pregnancy after systemic sclerosis diagnosis did not appear to significantly impact long-term renal, respiratory, or global function outcomes. While this offers a hopeful message to systemic sclerosis patients planning a pregnancy, physicians and patients should remain vigilant for potential post-partum complications.

## Introduction

Systemic sclerosis (SSc) is an autoimmune disease that can affect women during their reproductive years, yet there is limited research to inform women and healthcare providers about the effects of pregnancy on long-term disease activity and outcomes.^1^ Pregnancy induces many physiologic changes, including increased blood supply, fibrinogen levels, and immunological exposures.^2^ Peripartum maternal complications, such as hypertension of pregnancy and fetal complications, such as prematurity and low birth weights, have been described in short-term observational studies.^3^ Such studies highlight the limited data available to understand the longitudinal effects of pregnancy on SSc.

In a recent systematic review and meta-analysis, one or more SSc manifestations developed or worsened in 14.3% of women during their pregnancy and 10.5% of women in the 6 months post-partum. This included worsening overall disease activity (the term used in the review, but not clearly defined), (14.3% in pregnancy, 30% in the 6 months post-partum), Raynaud’s and/or digital ulcer (16.6% and 20.2%), gastrointestinal symptoms (15.3% and 11.7%), and arthritis (6.3% and 11.7%).^4^ Of the 1403 cases included, there were four maternal deaths secondary to renal crisis: congestive heart failure and aspiration pneumonia. Risk factors for obstetric complications include anti-topoisomerase I or anti-RNA polymerase III positivity,^5,6^ early diffuse cutaneous disease,^7^ and the use of corticosteroids.^8^

Studies to date have focused on SSc disease activity during pregnancy and first-year post-partum. However, little is known about the effects of pregnancy on disease activity in the extended post-partum period. We aim to address this gap using the Canadian Scleroderma Research Group (CSRG) database to retrospectively assess the disease progression of SSc in an extended post-partum period. We hypothesized that immunological and physiological changes during pregnancy may be associated with a more rapid disease progression of internal organ manifestations in the post-partum period, compared with nulliparous women.

## Methods

We used the data from the CSRG Database collected between 2004 and 2018. At the time of analysis, 1438 women were enrolled in the database. Of all female patients, 98% met the 2013 ACR/EULAR criteria.^9^ Women were included in the analyses if they were ⩾18 years of age and were either nulliparous or had at least one pregnancy following their diagnosis of SSc. We excluded women if they had a pregnancy before their SSc diagnosis and those who entered the cohort with comorbid cardiac, pulmonary, and renal disease unrelated to their diagnosis of SSc.

A descriptive analysis of baseline characteristics using means and standard deviations for continuous variables and frequencies/percentages for binary variables was performed. Student’s *t*-tests determined group differences for continuous variables and chi-square tests for categorical variables. Given that the outcomes of interest were changes in continuous variables over multiple periods of time, we used generalized estimating equations to analyze differences in indices of disease activity among cohorts. Data were collected annually for 1–10 years depending on how long the patient had been enrolled in the database. Outcomes examined included force vital capacity, diffusing capacity of the lungs for carbon monoxide (DLCO), right ventricular systolic pressure, glomerular filtration rate, physician global assessment of activity, and physician global assessment of severity. Each analysis was controlled for age, time since SSc diagnosis, SSc subtype (limited vs diffuse), and smoking status (pulmonary outcomes only).

Patients from all sites provided informed consent, and the research ethics board of each site approved the data collection protocol. Data analyses were performed using SASVR Studio software, version 3.8 for Windows.^10^

## Results

Within the CSRG database, 198 women met our inclusion criteria: 153 nulliparous women and 45 women with a pregnancy after their SSc diagnosis (Table 1). Of those excluded, 162 had pre-pregnancy cardiac conditions, 216 had chronic obstructive pulmonary disease, and 48 had kidney disease predating their diagnosis of SSc. Follow-up durations for women varied from 1 to 10 years (Table 2), with a median follow-up of 2.97 ± 2.94 years.

**Table 1.** Baseline characteristics on entrance to the CSRG database.

	Nulliparous, n = 152	Pregnancy after diagnosis, n = 45	*p* value
Age, years (mean, SD)	48.4 (13.6)	44.5 (8.0)	0.066
Age of diagnosis, years (mean, SD)	38.8 (14.2)	22.6 (6.8)	<0.001
Duration of disease, years (mean, SD)	9.6 (8.9)	21.9 (9.6)	<0.001
Smoker (n, %)	74 (49.3)	17 (37.8)	0.173
Meets ACR criteria (n, %)	149 (98.0)	43 (95.6)	0.355
Limited SSc (n, %)	94 (61.8)	30 (66.7)	0.556
Diffuse SSc (n, %)	58 (38.2)	15 (33.3)	0.610
Inflammatory arthritis present (n, %)	41 (27.0)	22 (48.9)	0.009
Myositis present (n, %)	17 (11.2)	7 (16)	0.440
Overlap present (n, %)	31 (20.4)	7 (16)	0.455
MCTD present (n, %)	8 (5.3)	3 (7)	0.7262
Raynaud’s present (n, %)	150 (98.7)	44 (98)	0.663
Ulcers present (n, %)	73 (48.0)	29 (64)	0.053
Scleroderma renal crisis (n, %)	2 (1.3)	2 (4)	0.194
History of malignancy (n, %)	10 (6.6)	2 (4)	0.593
CRP, mg/L (mean, SD)	3.7 (1.1–6.2)	2 (1.7–1.0)	0.375
ESR, mm/h (mean, SD)	17.5 (8.5–34.0)	9.5 (4.5–26.0)	0.311
Centromere positive (n, %)*	51 (41)	14 (35)	0.514
Topoisomerase positive (n, %)*	23 (18)	5 (13)	0.387
RNA pol III positive (n, %)*	16 (13)	1 (3)	0.062
eGFR, mL/min/1.73 m^2^ (mean, SD)	155.4 ± 48.6	157.0 ± 53.6	0.858
DLCO, mL/min/mmHg (mean, SD)	74.9 ± 22.6	75.9 ± 20.8	0.823
FVC, % predicted (mean, SD)	90.2 ± 19.1	91.0 ± 18.8	0.837
FEV1/FVC (mean, SD)	79. 7 ± 9.9	78.8 ± 8.6	0.658
RVSP, mmHg (mean, SD)	34.5 ± 15.1	31.5 ± 7.0	0.426

SD: standard deviation.

* Data are missing for 32 women.

**Table 2.** Number of patients with follow-up data at each year in the CSRG database.

Years of follow-up	Nulliparous	PAD of SSc
Entry into registry	153	45
1 year	132	41
2 years	110	39
3 years	93	34
4 years	73	29
5 years	58	25
6 years	48	21
7 years	35	14
8 years	26	12
9 years	18	9

PAD: pregnancy after systemic sclerosis diagnosis.

Across baseline characteristics, nulliparous women were diagnosed with SSc at an older age (38.7 versus 22.6 years; *p* < 0.001), had a shorter duration of disease (9.6 versus 21.9 years; *p* < 0.001) and more arthritis at baseline (41 versus 22; *p* = 0.009). There was a trend toward more RNA polymerase III antibody positivity in nulliparous women (16 versus 1; *p* = 0.06), although this was not statistically significant. There was no difference in other findings, including subtypes of SSc and anti-topoisomerase positivity.

In the pregnancy cohort, the mean age of conception was 29.5 years of age. Women had, on average, 2.1 ± 1.1 pregnancies, 1.6 ± 0.9 live births, 0.4 ± 0.9 miscarriages, and 0.1 ± 0.4 therapeutic abortions.

Using the generalized estimating equations, pregnancy was not found to affect markers of disease progression, including force vital capacity (*p* = 0.90), DLCO (*p* = 0.62), right ventricular systolic pressure (*p* = 0.31), estimated glomerular filtration rate (*p* = 0.43), physician global assessment of activity (*p* = 0.69), and physician global assessment of severity (*p* = 0.75).

## Discussion

Pregnancy does not appear to impact the internal organ manifestations of SSc over an extended post-partum period. Compared to previous studies which were limited to 1 year of post-partum data, this study examined annual data in an extended post-partum period, up to 10 years, to assess for any long-term disease complications that may be related to the physiologic stress of pregnancy. Physiologic changes in pregnancy could potentially exacerbate SSc-related organ involvement such as pulmonary hypertension, congestive heart failure, ventricular arrhythmias, and gastroesophageal reflux. In our cohort, we did not see an increase in the prevalence of renal disease, pulmonary hypertension, or interstitial lung disease, compared to nulliparous women.

When comparing the baseline characteristics of our two cohorts, the nulliparous cohort had a lower prevalence of inflammatory arthritis (28%) compared to the pregnancy cohort (49%). The rate of inflammatory arthritis in the nulliparous cohort is also lower than the prevalence of inflammatory arthritis in general SSc cohorts, which is reported at 31%–61%.^11–13^ While the prevalence seen in nulliparous women may be a result of a small sample size, it could also be related to breaking immune tolerance during pregnancy.

Our results are encouraging for women with SSc who wish to become pregnant. We had expected to see evidence of worsening disease following pregnancy, as has been observed in other autoimmune spectrum conditions. For example, a systematic review found that 47% of women with rheumatoid arthritis experienced an increase in flares post-partum compared to their rate of flares during pregnancy.^14^ A Norwegian registry for patients with systemic lupus erythematosus found a significant increase in disease activity at 6 months and 12 months post-partum.^15^ Castellino et al.^16^ found that 12% of women with an undifferentiated connective tissue disease (CTD) before pregnancy progressed to a well-defined CTD post-partum, compared to 2% in a non-pregnant control group. A retrospective case–control study reported that 10% of women with Sjogren’s syndrome flared 1-year post-partum.^17^ These studies were limited to a 1-year follow-up, and it is possible that pregnancy may have only a short-term impact on the trajectory of autoimmune diseases.

Anti-topoisomerase positivity was identified as a possible risk factor for post-partum disease progression in the IMPRESS study, a 1-year prospective study of maternal outcomes in SSc patients compared with the general obstetric population. The IMPRESS study reported progressive internal organ damage in 4/109 women in their first-year post-partum, all of whom were anti-topoisomerase positive. Our nulliparous group had a slightly higher prevalence of anti-topoisomerase positivity than the pregnancy group (13% in the pregnancy cohort, 18% in the nulliparous cohort, *p* = 0.387). In contrast, our prevalence of anti-topoisomerase positivity is notably lower than the 54% prevalence reported in the IMPRESS study. If anti-topoisomerase positivity is an important risk marker, it is likely that our study was under-powered to detect significant group differences. Anti-topoisomerase is also more frequently associated with diffuse SSc, and the rates of diffuse SSc were also somewhat higher in the IMPRESS cohort (48%) compared with the CSRG database (38.2% in the pregnancy cohort and 33.3% of nulliparous cohort). These may have been predisposing factor for more severe visceral organ disease progression in the IMPRESS study, which was not seen in our study.

Our study has several limitations. The pregnancy cohort was relatively small, and only a small portion of women had 10 years of data in the registry. Survivorship bias may have been introduced because women with less-severe disease may have been more likely to conceive after their SSc diagnosis, compared with those who presented with extensive disease. Also, women in the pregnancy cohort had a markedly longer disease duration at entry into the database compared with nulliparous women, raising the possibility that women with poor pregnancy outcomes and/or decreased survival may not have been captured in the database.

We were also limited by the registry data, which did not specify pregnancy dates. In the absence of this information, we were unable to assess the effects of pregnancy on disease progression in the immediate post-partum period. The more time that passes after pregnancy, the higher the likelihood that other factors may contribute to disease progression, and the less confident we can be of a causal link between pregnancy and disease trajectory.

While acknowledging its limitations, this retrospective cohort analysis suggests that having ⩾1 pregnancy after a diagnosis of SSc may not significantly impact renal, respiratory, or global function over 10 years of follow-up. While our study offers a hopeful longer-term message for patients with scleroderma who are planning a pregnancy, physicians and patients should remain vigilant for post-partum complications.
